# Analysis of FDA's Accelerated Approval Program Performance December 1992–December 2021

**DOI:** 10.1007/s43441-022-00430-z

**Published:** 2022-07-28

**Authors:** Ginny Beakes-Read, Madison Neisser, Patrick Frey, Mara Guarducci

**Affiliations:** Global Regulatory and R&D (GRR&D) Policy, Amgen, Inc., 601 13th Street NW Suite 1100 North, Washington, DC 20005 USA

**Keywords:** Accelerated approval, Conversion, Not yet converted, Ongoing studies, Confirmatory trials, Dangling approvals

## Abstract

The accelerated approval pathway has been criticized recently for employing lower regulatory standards than traditional drug approval, undue delays in withdrawing approvals of drugs for which studies have not confirmed clinical benefit, and confirmatory trials not being pursued with due diligence. This article examines the status of confirmatory studies of drugs approved under the US Food and Drug Administration’s (FDA’s) accelerated approval program between December 1992 and December 2021. It includes background on the program and provides broader context about the program’s performance to date over its 30-year history. Our analysis demonstrates that the accelerated approval program has been largely successful, with half of accelerated approvals converted to traditional approval in a median time of 3.2 years. Furthermore, recent FDA actions show that the agency is appropriately managing the program when a drug approved under accelerated approval fails to confirm a clinical benefit. Any proposed changes to the program should be based on cumulative experience with the program, rather than outliers.

## Introduction

Accelerated approval was established in 1992 by US Food and Drug Administration (“FDA”) regulation in response to the AIDS epidemic to address the urgency for life-saving treatments. Codified in statute by Congress in 2012, the pathway “accelerates” access to medicines for serious and life-threatening conditions because approval can be granted earlier, based on a surrogate endpoint [1] that is reasonably likely to predict clinical benefit, rather than waiting until an effect on irreversible morbidity or mortality or other clinical benefit can be demonstrated.

FDA applies the same evidentiary standard for an accelerated approval that the agency must use for a traditional approval [2]. An accelerated approval from FDA means the agency concluded that the drug is safe and effective for the particular indication for use while acknowledging that there is uncertainty whether the surrogate endpoint will correlate to an ultimate clinical benefit for that indication [3]. Accepting more uncertainty is the trade-off for faster access to important therapies for patients with serious and life-threatening disease.

Following an accelerated approval, sponsors usually must verify that the drug has an effect on irreversible morbidity or mortality or other clinical benefit. This is done through confirmatory clinical studies that often begin prior to the approval, consistent with FDA guidance issued in 2014 [4]. At the time of approval, FDA and sponsors agree on target timelines for completion of study milestones, such as interim reports, study completion, and submission of the final report. FDA includes these milestone dates, along with study requirements, in the approval letter and reports the status on FDA’s website [5]. This public tracking helps all stakeholders monitor progress of the confirmatory study requirements.

Sometimes, the initial milestones cannot be met for various reasons, for example, the inability to enroll subjects at the expected rate or the fact that a study is dependent on the results of another study that has been delayed. In such cases, if the sponsor is still actively conducting the study, the sponsor may work with FDA to revise certain milestones. FDA provides updated information on its website but does not routinely post new dates for the milestones.

When FDA has received the final study report for the confirmatory study (or studies) and agrees that it confirms benefit, FDA will convert the accelerated approval to a traditional approval. If the study does not confirm benefit or is unreasonably delayed, FDA may ask the sponsor to voluntarily withdraw the drug from the market or remove the particular indication for the drug from its labeling. If the sponsor does not voluntarily withdraw the drug or indication, FDA can initiate withdrawal proceedings and ultimately withdraw the approval [6].

Drugs for which confirmatory trials have been conducted that did not confirm clinical benefit but that remain on the market have been described as dangling approvals [7]. FDA says that it is “continuously evaluating these approvals to ensure the safety and effectiveness of approved products and whether a ‘dangling’ accelerated approval indication should remain approved.” [7]. In such cases, FDA usually discusses with the sponsor the reasons the study did not confirm benefit and will explore alternative trial designs if an unmet medical need or conditions for accelerated approval still exist. Drugs for which the confirmatory trials have missed the original and any revised milestones are considered “delinquent.” (“Dangling” and “delinquent” are informal terms used by FDA’s Oncology Center of Excellence; those terms are not used on the Agency’s Postmarket Requirements and Commitments website.) [5].

## Materials and Methods

This article examines the status of drugs approved under the FDA’s accelerated approval program between December 1992 and December 2021 and the status of confirmatory studies of those drugs. The research and findings were conducted and summarized by Amgen and are based on FDA source material including CDER’s Accelerated Approvals report [8], the FDA Postmarket Requirements and Commitments website [5], as well as review documents posted on the FDA-Approved Drugs site, Drugs@FDA [9].

Drugs are approved for specific indications for use which are reflected in the drugs’ labeling. Accelerated approval may apply to the drug and all its indications or only to specific indications. When a drug is approved for a single indication, if the confirmatory study for the indication does not confirm clinical benefit, then the drug may be completely withdrawn from the market. If it is approved for multiple indications, the affected indications can be removed from the labeling individually, leaving the drug on the market for indications approved under traditional approval or for which post-marketing studies are still under way. In the research findings below, the term “accelerated approval” may refer to accelerated approval of a drug with a single indication or the approval of certain indications of a drug with multiple indications. Therefore, the same drug may be reflected in the statistics more than once.

## Results

### Accelerated Approvals: December 1992–December 2021

Over the 30-year life of the accelerated approval program, FDA has approved 278 drugs under accelerated approval. In the first 10 years of the program, 65% of FDA’s accelerated approvals were for infectious diseases, a figure consistent with the original intent of the program to expedite the availability of HIV treatments. In the second two decades, FDA increasingly used the pathway to help bring oncology drugs to market. In the last 10 years, 2012–2021, 83% of accelerated approvals have been for oncology (including hematology–oncology) indications [10]. During this time, there was an increase in development of targeted therapies in oncology that, along with the availability of more well-established oncology surrogate endpoints, contributed to the use of the accelerated approval pathway [11].

**Fig. 1:**
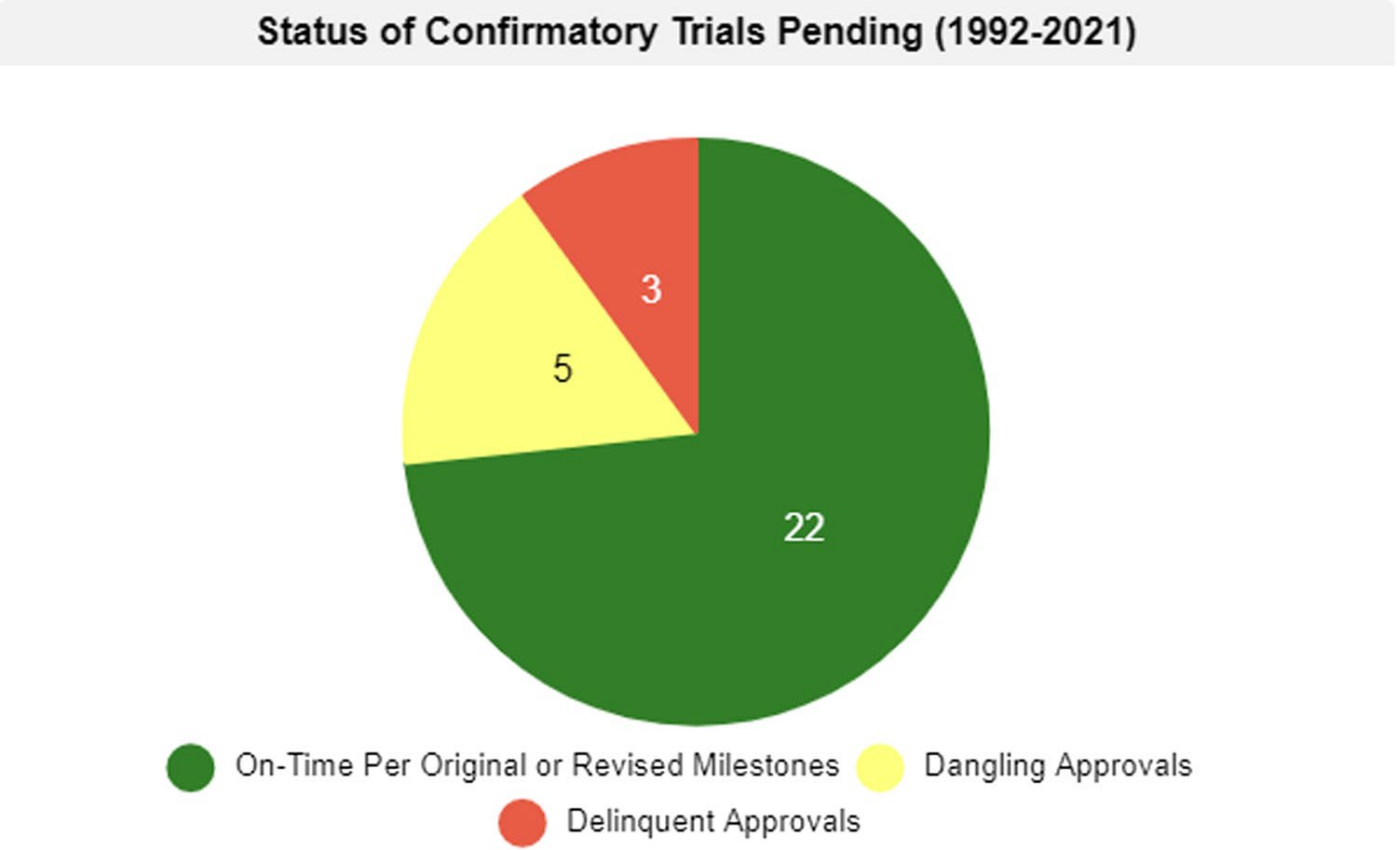
Accelerated approvals pending longer than 3.2 years by category cohort [5]

Accelerated approvals generally fit into one of three categories: converted to traditional approval, withdrawal of approval, or confirmatory trials still pending completion or FDA review (Tables 1, 2; Fig. 1).

**Table 1:** Accelerated approvals by decade cohort: converted, withdrawn, pending completion [5]

1992–2001 summary data		2002—2011 summary data		2012—2021 summary data	
Total accelerated approvals granted	52	Total accelerated approvals granted	59	Total accelerated approvals granted	167
Converted	44	Converted	44	Converted	51
Confirmatory trials pending	2	Confirmatory trials pending	3	Confirmatory trials pending	102
Of 44 converted, average time to confirm clinical benefit was 4.5 years, with a median of 3.9 years		Of 44 converted, average time to confirm clinical benefit was 5 years, with a median of 4.1 years		Of 51 converted, average time to confirm clinical benefit was 2.5 years, with a median of 2.3 years	
Of 6 withdrawn, the average time to withdrawal was 8.9 years, with a median of 10.4 years		Of 12 withdrawn, the average time to withdrawal was 9.9 years, with a median of 9.7 years		Of 14 withdrawn, the average time to withdrawal was 3.7 years, with a median of 3.5 years

**Table 2:** Status of accelerated approvals pending longer than 3.2 years by calendar cohort [8]

1992–2001 confirmatory trials pending		2002–2011 confirmatory trials pending		2012–2021 confirmatory trials pending	
On-time per original or revised milestones	0	On-time per original or revised milestones	2	On-time per original or revised milestones	20
Dangling approval	1	Dangling approval	1	Dangling approval	3
Delinquent approval	1	Delinquent approval	0	Delinquent approval	2

### Conversion of Accelerated Approvals to Traditional Approvals

As of December 31, 2021, FDA has converted 50% of accelerated approvals (139) to traditional approval based on studies that have confirmed clinical benefit. For these conversions, the median time from accelerated approval to traditional approval was 3.2 years. In the last decade, 51 of the accelerated approvals were converted in a median time of 2.3 years. (This number will change as the cohort continues to convert.) During this period of faster conversion to traditional approval, in 2014, FDA also finalized its guidance on expedited programs [4]. This guidance affirmed the agency’s expectation that, at the time of accelerated approval, either confirmatory trials be underway, or FDA and sponsors have reached agreement on the final protocol for the confirmatory study.

### Withdrawals of Approval

As of December 31, 2021, 12% of accelerated approvals (32) have been withdrawn, either voluntarily by the sponsor or involuntarily after FDA proceedings [8]. For accelerated approvals granted between 1992 and 2001 (6 withdrawals), the median time from the date of initial approval of the application to the date of withdrawal was 10.4 years [8]. For accelerated approvals granted between 2002 and 2011 (12 withdrawals), the median time from the date of initial approval of the indication to the date of withdrawal was 9.7 years [8]. In the most recent decade between 2012 and 2021 (14 withdrawals), the median time between approval and the date of withdrawal was 3.5 years [8]. This is a significant improvement over past decades, in part because of proactive efforts FDA has undertaken. For example, FDA has used the Oncologic Drug Advisory Committee (ODAC) meetings to identify and publicly discuss dangling accelerated approvals. These meetings help FDA to assess whether there is a path forward to confirm clinical benefit remains or whether withdrawal of the accelerated approval may be the appropriate action.

### Confirmatory Trials Still Pending Completion or FDA Review

As of December 31, 2021, 38% of accelerated approvals (107) have not been converted to traditional approval, pending completion and FDA review of confirmatory trials. 77 have been marketed for less than 3.2 years (less than the current median time from date of approval to date of conversion). Because this can be seen as a reasonable period in which confirmatory trials could be expected to be completed, this cohort is excluded from further analysis. That leaves 30 accelerated approvals to evaluate. Those 30 approvals are pending completion and FDA review of confirmatory trials and have been marketed for more than 3.2 years. These pending accelerated approvals are the focus of the rest of this article because they have not yet converted to traditional approval within this median 3.2-year timeline.

### Status of 30 Accelerated Approvals Pending Longer than 3.2 Years

Of the 30 accelerated approvals that are pending completion and FDA review of confirmatory trials for longer than 3.2 years, 22 are considered to be on-time, that is, they are proceeding in accordance with, or ahead of, the original or revised milestones. 10 are on-time according to the original milestones and 12 are on-time according to revised milestones. 5 of the accelerated approvals pending longer than 3.2 years without conversion are “dangling” approvals and 3 are “delinquent.”

### Revised Milestones

#### Of the 12 with Revised Milestones

- In 3 cases in the 2012–2021 cohort, milestones were revised because of the COVID-19 pandemic (e.g., difficulties completing trials, challenges with enrollment, or trial start-up).
- In 2 cases, milestones were revised because the start of the trial was dependent on results from another study that was delayed.
- In 4 cases (all oncology), milestones were revised to accumulate more data. In these cases, the number of progression-free survival (PFS) events that guided expected trial timelines did not occur as quickly as anticipated.
- In 1 case, FDA acknowledged revised milestones because additional time was needed to revise the study protocol and statistical analysis plan based on Agency feedback.
- In 1 case, the final protocol submission date was revised. No additional information is provided on the FDA website.
- In 1 case, the Agency requested submission of a revised protocol, which has since been submitted.

### Dangling Approvals

In the 5 “dangling” cases, FDA is evaluating whether the products or indications should remain on the market.

- In 1 case for a drug with accelerated approval that is now marketed only as a generic, the requirement to verify the clinical benefit remains. The referenced innovator product was withdrawn from the market, but not for safety or efficacy reasons [12].
- In 3 cases, the reasons given for the confirmatory trial not verifying clinical benefit were enrollment challenges in the trial or a change in the treatment landscape rendering the trial infeasible. FDA indicated that there are alternative options to confirm clinical benefit.
- In 1 recent case, FDA has initiated procedures to withdraw the accelerated approval and granted a public hearing (not yet scheduled) [13].

### Delinquent Approvals

- In 1 delinquent case, a confirmatory study is not yet started due to ongoing discussion with FDA about study design. The other 2 delinquent cases involve a missed milestone deadline for submission of the final protocol in one case and a missed milestone deadline for submission of the final study report in the other.

## Discussion

The accelerated approval pathway is working effectively to accelerate patient access to treatments for serious or life-threatening diseases or conditions, sometimes when no other therapy exists. Concerns voiced recently about the program are based on a small number of examples, and do not reflect overall management of the accelerated approval program over its 30-year history, particularly improvements seen in the last decade in the time from accelerated approval to conversion. Clinical benefit is confirmed in a vast majority of cases (accelerated approval is converted to traditional approval), evidence that sponsors and FDA are managing the program appropriately. The small percentage of drugs whose clinical benefit is ultimately not confirmed should not be viewed as a failure of the accelerated approval program. Rather, they represent an expected trade-off in expediting drug development that benefits patients with serious or life-threatening diseases [14].

Although there are studies pending completion and FDA review that extend beyond the current 3.2 years median time to conversion, FDA continues to acknowledge the need for these ongoing trials by revising milestones and actively monitoring progress. FDA revises study milestones when, for example, challenges arise during the conduct of a trial. In the case of dangling approvals, FDA is proactively addressing the issues. When it is clear that the trial will not meet its goal, FDA views discussions with sponsors and other stakeholders on dangling approvals as an essential step to determine if an unmet medical need or conditions for accelerated approval still exist before additional confirmatory trials are explored [15]. For example, as a result of these discussions, four dangling approvals were voluntarily withdrawn between 2020 and 2021 [8]. FDA also convened an ODAC meeting in 2021 to address six dangling approvals [16]. Following the meeting, four additional dangling approvals were voluntarily withdrawn in 2021 [8]. Another ODAC meeting scheduled later that same year was subsequently canceled due to an additional voluntary withdrawal of an accelerated approval indication [8].

## Conclusion

Over the 30-year history of the accelerated approval program, there have been relatively few delinquent cases or dangling approvals. Legislative or other changes to the program should be based on cumulative experience, not outliers. Any changes must be carefully crafted to avoid creating disincentives that would reduce access to life-saving treatments under the accelerated approval pathway. FDA has demonstrated the ability to manage challenges with the program through employing tools currently available to the Agency. For example, FDA has issued guidance that sponsors and FDA should either reach agreement on the final protocol for the confirmatory study or have the study underway at the time of accelerated approval [4]. Additionally, FDA has used the Advisory Committee process to highlight issues and challenges with particular accelerated approvals, resulting in voluntary withdrawals in many cases. To the extent that increased public confidence in the program is needed, additional transparency about the status of post-marketing studies could address that concern. FDA should share publicly on its database more information when a postmarket clinical study milestone is revised, including posting revised milestone dates, as well as a more detailed explanation for extending a study deadline. FDA should include why FDA remains confident in the sponsor’s ability to complete the confirmatory study.
